# Outcomes of left bundle branch area pacing compared to His bundle pacing and right ventricular apical pacing in Japanese patients with bradycardia

**DOI:** 10.1002/joa3.12997

**Published:** 2024-01-28

**Authors:** Hiroyuki Kono, Shoichi Kuramitsu, Masato Fukunaga, Kengo Korai, Michio Nagashima, Kenichi Hiroshima, Kenji Ando

**Affiliations:** ^1^ Department of Cardiology Kokura Memorial Hospital Kitakyushu Japan; ^2^ Department of Cardiovascular Medicine Sapporo Cardiovascular Clinic, Sapporo Heart Center Sapporo Japan

**Keywords:** atrioventricular block, heart failure, His bundle pacing, left bundle branch area pacing, right ventricular apical pacing

## Abstract

**Background:**

His bundle pacing (HBP) and left bundle branch area pacing (LBBAP) emerge as better alternatives to right ventricular apical pacing (RVAP) in patients with bradycardia requiring permanent cardiac pacing. We aimed to compare the clinical outcomes of LBBAP, HBP, and RVAP in Japanese patients with bradycardia.

**Methods:**

A total of 424 patients who underwent successful pacemaker implantation (HBP, *n* = 53; LBBAP, *n* = 75; and RVAP, *n* = 296) were retrospectively enrolled in this study. The primary study endpoint was the cumulative incidence of heart failure hospitalization (HFH) during the follow‐up.

**Results:**

The success rate for implantation was higher in the LBBAP group than in the HBP group (94.9% and 81.5%, respectively). Capture threshold increase >1V during the follow‐up occurred in the HBP and RVAP groups (9.4% and 5.1%, respectively), while it did not in the LBBAP group. The cumulative incidence of HFH was significantly lower in the LBBAP group than the RVAP (adjusted hazard ratio, 0.12 [95% confidence interval: 0.02–0.86]; *p* = .034); it did not differ between the HBP and RVAP groups (adjusted hazard ratio, 0.48 [95% confidence interval: 0.17–1.34]; *p* = .16). Advanced age, mean percent right ventricular pacing (per 10% increase), left ventricular ejection fraction <50%, and RVAP were associated with HFH.

**Conclusions:**

Compared to RVAP and HBP, LBBAP appeared more feasible and effective in patients with bradycardia requiring permanent cardiac pacing.

## Graphical abstract

A total of 424 patients were analyzed, including 296 with right ventricular apex pacing, 53 with His bundle pacing, and 75 with left bundle branch area pacing, with heart failure hospitalization as the endpoint. Compared to right ventricular apical pacing and His bundle pacing, left bundle branch area pacing appeared more feasible and effective in patients with bradycardia requiring permanent cardiac pacing.

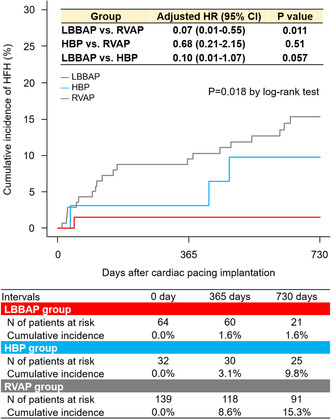

## 1 INTRODUCTION

Permanent cardiac pacing is the most effective treatment for patients with chronic high‐degree atrioventricular block (AVB) and symptomatic sick sinus syndrome. Right ventricular apical pacing (RVAP) has been widely used for these patients. However, RVAP causes electronic and mechanical dyssynchrony, associated with an increased risk of heart failure.^1^ Although different right ventricular (RV) site (e.g., the septum and outflow tract) pacing had been attempted to overcome these limitations, meta‐analyses have not demonstrated the superiority of alternative RV pacing to RVAP.^2^, ^3^

In recent years, conduction system pacing (CSP), a technique directly activating His bundle or left bundle branch, has attracted attention as a novel technique.^4^, ^5^, ^6^, ^7^, ^8^, ^9^, ^10^ His bundle pacing (HBP) has demonstrated its feasibility and clinical benefits compared with RAVP.^4^, ^5^ Despite these advantages, HBP has some limitations in terms of technical aspects (e.g., relatively lower procedural success rates and a high and unstable pacing threshold in 5% to 10% of patients).^4^, ^5^ On the other hand, left bundle branch area pacing (LBBAP) emerges as a new physiological pacing therapy that can generate a comparably narrow‐paced QRS complex and left ventricular activation with a low, stable pacing threshold.^6^ The collective term “LBBAP” encompasses left bundle branch pacing (LBBP), left fascicular pacing (LFP), and left ventricular septal pacing (LVSP).^7^ This categorization arises from challenges in differentiation and the limited clinical data highlighting their differences. Previous studies reported that LBBP was superior to LVSP in enhancing pacing characteristics, such as LVAT and QRS duration.^8^, ^9^ LVSP was not part of the success criteria for LBBAP in initial studies,^10^, ^11^, ^12^, ^13^, ^14^, ^15^, ^16^, ^17^, ^18^, ^19^ while the latest consensus document has included LVSP for LBBAP.^7^ Recent clinical studies have demonstrated that LBBAP led to better outcomes than RVAP^10^, ^11^; LBBAP boasted a higher success rate and a more consistent pacing capture threshold than HBP.^13^, ^14^, ^15^, ^16^, ^17^, ^18^, ^19^, ^20^ Although previous studies compared clinical outcomes between LBBAP and HBP, little data are available regarding the feasibility and efficacy of LBBAP in Japanese subjects. Therefore, the current study sought to investigate the clinical outcomes of LBBAP compared with HBP and RVAP in Japanese patients with bradycardia requiring permanent cardiac pacing.

## 2 METHODS

### 2.1 Study population

This was a retrospective, observational study at Kokura Memorial Hospital, Kitakyushu, Japan. From June 2018 and June 2021, a total of 481 consecutive patients underwent de novo permanent pacemaker implantation for bradycardia indications.^21^ In the present study, we retrospectively sought to enroll patients undergoing RVAP, HBP, and LBBAP among them. The choice of pacing technique (i.e., RVAP, RV septum pacing, HBP, or LBBAP) was determined based on the operator's professional judgment. Patients with (1) ≤20 years old, (2) RV septum pacing, (3) leadless pacemaker implantation, (4) implantable cardioverter defibrillator implantation, or (5) cardiac resynchronization therapy were excluded. The research protocol received approval from the ethics committee at Kokura Memorial Hospital and adhered to the principles outlined in the Declaration of Helsinki. Written informed consent was waived because of the retrospective study design. This study was registered with http://www.umin.ac.jp, unique identifier UMIN000053252.

### 2.2 Procedures

Right ventricular apical pacing was performed in a standard fashion; RV leads were implanted at the RV apex using a hand‐shaped style. HBP and LBBAP were performed using the Select Secure (model 3830, 69 cm, Medtronic, Inc., Minneapolis, Minnesota) pacing lead delivered through a fixed curve sheath (C315HIS, Medtronic) as previously reported.^4^, ^5^, ^6^, ^7^, ^10^, ^11^ HBP was utilized to position the sheath at the base of the tricuspid annulus in the right ventricle. Screw‐in was performed at the location where the His bundle potential was detected on the unipolar lead. A successful HBP was determined by the detection of the His bundle potential without surpassing a pacing capture threshold of 3.5 V/0.4 ms or 3 V/1 ms. If failed, RV septum pacing was undergone. We defined selective HBP when the QRS waveform appeared with latency from the pacing spike on the 12‐lead ECG and nonselective HBP when it did not.^22^

On the other hand, LBBAP involved positioning the sheath 1.5–2 cm distal to the His bundle in preparation for screw‐in. When targeting the left ventricular septum, we monitored the pacing waveform in V1 lead and observed a transition from “W” waveform to “rSR” or “QR.” For depth verification, contrast was injected through the sheath. The R‐wave peak time in V6 (V6RWPT) was assessed in a 12‐lead electrocardiogram just after the procedure.^7^ Notably, V6RWPT represented the interval from the onset of the pacing spike to the peak of the R wave in the V6 lead.^22^ Successful LBBAP was characterized by the emergence of R waveforms such as “rSR,” “qR,” “QR,” and “Qr” in the V1 lead. Moreover, if the intrinsic QRS was either normal or right bundle branch block (RBBB), V6RWPT of ≤85 ms was required. In instances where the intrinsic QRS had conduction disturbances other than RBBB, V6RWPT of ≤100 ms was necessary. For a specific LBBP classification, the V6RWPT was defined as ≤75 ms if the intrinsic QRS was normal or RBBB, or ≤80 ms if other conduction disturbances were evident.^7^ Patients who did meet the criteria of LBBP were categorized as LVSP. In cases where LBBAP was unsuccessful, RV septum pacing was performed.

### 2.3 Data collection and follow‐up

Electrical performance (e.g., pacing capture thresholds, pacing impedances, sensed R‐wave amplitudes, and %RV pacing) was assessed at outpatient visits at 1, 3, 6, and 12 months and then yearly postimplantation or via remote monitoring devices when feasible. Percent RV pacing was determined at the end of follow‐up, censored to an earlier date if the primary outcomes occurred. Lead‐related complications, including infection, dislodgement, loss of capture, and early battery depletion, were also tracked over the follow‐up. Delta QRS duration means the difference between paced QRS duration and intrinsic QRS duration. Clinical follow‐up data were systematically gathered from medical records or via telephone contact with the patients, their families, or referring physicians. Pacing‐induced cardiomyopathy (PICM) was defined as left ventricular ejection fraction (LVEF) <40% or a necessity for biventricular pacing (BVP) upgrade.^23^

### 2.4 Study endpoints and definitions

The primary study endpoint was the cumulative incidence of heart failure hospitalization (HFH) during the follow‐up. HFH was defined as an unplanned hospitalization for worsening heart failure or de novo heart failure events.^24^ The secondary endpoints included (1) all‐cause death, (2) cardiac death, (3) a need for BVP upgrade; and (4) major cardiovascular events (MACE; a composite of all‐cause death, HFH, and a need for BVP upgrade). Also, we assessed the cumulative incidence of HFH between the CSP (LBBAP and HBP) and RVAP groups. Death was regarded as cardiac death unless other noncardiac death could be identified.

### 2.5 Statistical analysis

Data are presented as median (lower and upper qualities) for continuous variables and number (percentage) for categorical variables. Group comparisons were performed by the one‐way analysis of variance or Kruskal‐Wallis test for continuous variables, the chi‐square test, or Fisher's exact test for categorical variables, as appropriate. Cumulative incidence rates of study endpoints were estimated using Kaplan–Meier curves and compared by the log‐rank test. Hazard ratios (HRs) with 95% confidence intervals (CIs) of (1) the LBBAP group relative to the RVAP and HBP groups and (2) the HBP group relative to the RVAP group for the outcome measures were estimated throughout the entire follow‐up period by a multivariable Cox model. Multivariable models adjusted for the clinically relevant variables as follows: age, male gender, body mass index, AVB, hypertension, dyslipidemia, diabetes mellitus, LVEF <50%, hemodialysis, prior arterial fibrillation, prior coronary artery graft bypass, prior percutaneous coronary intervention, prior HFH, and percent RV pacing. Finally, we sought to identify potential risk factors for HFH by applying the multivariable Cox models, including 5 clinically relevant covariates (pacing mode [HBP, LBBAP, and RVAP], age, prior atrial fibrillation, LVEF [<50% or not], and mean %RV pacing).^1^, ^22^, ^25^

Statistical analyses for the study were conducted by two physicians (Drs Kono and Kuramitsu) utilizing R software version 3.5.2 (R Foundation for Statistical Computing, Vienna, Austria). A two‐sided *p*‐value of less than .05 was deemed to indicate statistical significance.

## 3 RESULTS

### 3.1 Study population

**FIGURE 1:**
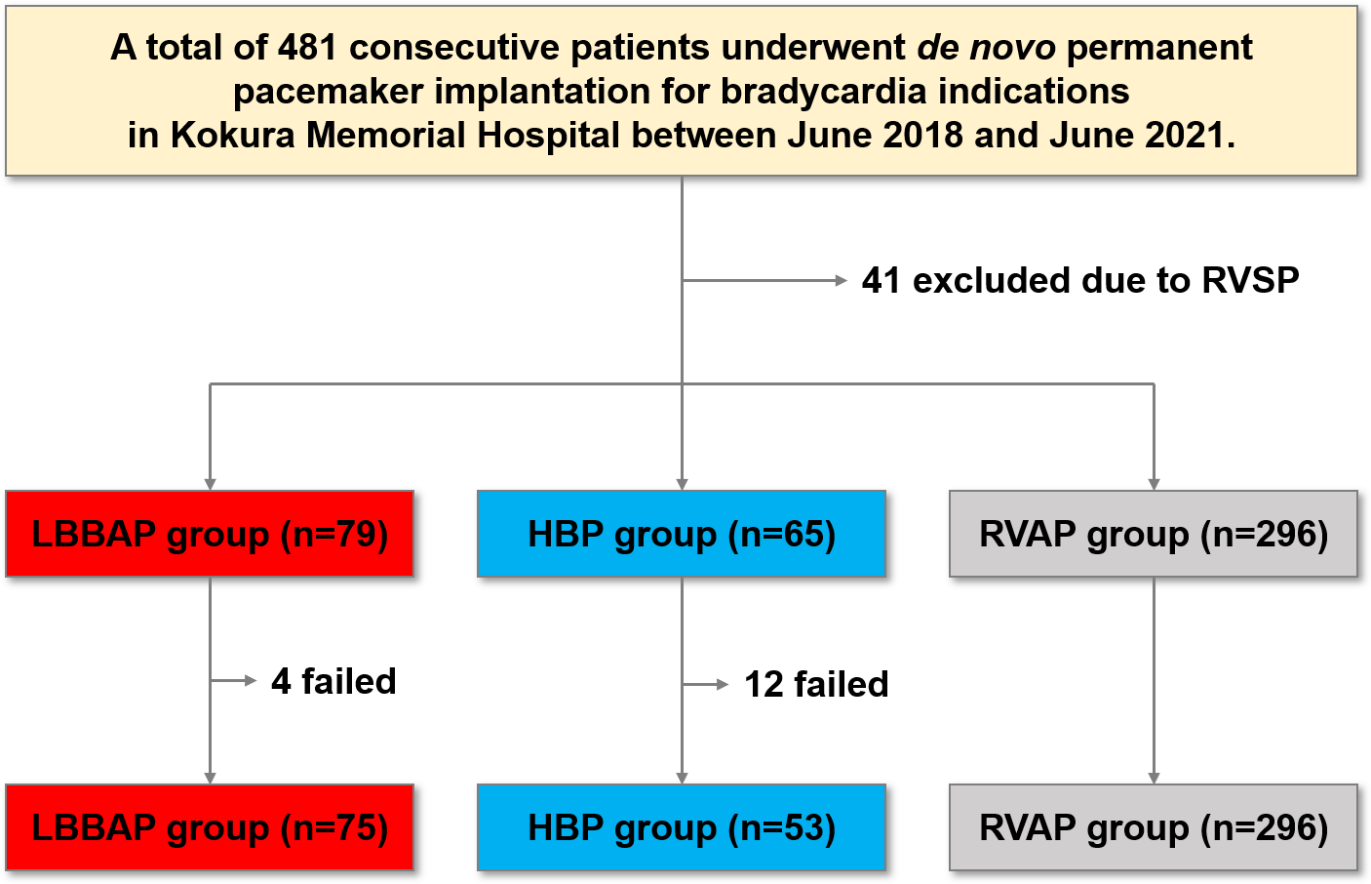
Study flowchart. RVSP, right ventricular septal pacing. Other abbreviations as in Table 1.

Of the 481 patients, 57 were excluded due to the following reasons: RV septum pacing (*n* = 41), failed HBP (*n* = 12), and failed LBBAP (*n* = 4). Finally, 424 (LBBAP, *n* = 75; HBP, *n* = 53; RVAP, *n* = 296) were analyzed in this study (Figure 1). The procedural success rate was higher in the LBBAP group than in the HBP group (94.9% and 81.5%, respectively). The reasons for the failed HBP and LBBAP were uncaptured His potential and hardness of septum, respectively.

### 3.2 Baseline clinical characteristics

The baseline clinical characteristics are summarized in Table 1. Compared with the LBBAP and HBP groups, the RVAP group had a higher prevalence of prior atrial fibrillation (32.1% vs. 13.3% vs. 20.8%, *p* = .003) and a lower prevalence AVB (52.4% vs. 81.3% vs. 60.3%, *p* < .001). Although the LBBAP group had a higher BNP value than the HBP and RVAP groups (205.7 pg/dL [67.1, 466.7] vs. 103.8 pg/dL [45.2, 218.5] vs. 153.0 pg/dL [69.7, 444.3], *p* = .017), prior HFH, LVEF, and medication at discharge did not differ among the three groups.

**TABLE 1:** Baseline clinical characteristics.

	Overall (*n* = 424)	LBBAP (*n* = 75)	HBP (*n* = 53)	RVAP (*n* = 296)	*p* Value
Age, years	78.0 (72.0, 84.0)	78.0 (71.0, 86.0)	79.0 (73.0, 83.0)	78.0 (71.0, 83.0)	.65
Male sex	222 (52.4%)	39 (52.0%)	23 (43.4%)	160 (54.1%)	.36
BMI, kg/m^2^	23.1 (21.0, 25.4)	24.2 (21.3, 26.4)	23.2 (21.0, 25.3)	22.9 (20.9, 25.1)	.14
Hypertension	282 (66.5%)	50 (66.7%)	41 (77.4%)	191 (64.5%)	.19
Dyslipidemia	182 (42.9%)	41 (54.7%)	33 (62.3%)	108 (36.5%)	<.001
Diabetes mellitus	115 (27.1%)	23 (30.7%)	19 (35.8%)	73 (24.7%)	.18
Hemodialysis	16 (3.8%)	1 (1.3%)	2 (3.8%)	13 (4.4%)	.46
Prior PCI	61 (14.4%)	14 (18.7%)	6 (11.3%)	41 (13.9%)	.45
Prior CABG	16 (3.8%)	2 (2.7%)	2 (3.8%)	12 (4.1%)	.85
Prior atrial fibrillation	116 (27.4%)	10 (13.3%)	11 (20.8%)	95 (32.1%)	.003
Prior heart failure hospitalization	32 (7.5%)	6 (8.0%)	2 (3.8%)	24 (8.1%)	.54
Prior stroke	52 (12.3%)	8 (10.7%)	7 (13.2%)	37 (12.5%)	.89
Clinical presentation					
Atrioventricular block	248 (58.5%)	61 (81.3%)	32 (60.3%)	155 (52.4%)	<.001
Sick sinus syndrome	165 (38.9%)	13 (17.3%)	20 (37.7%)	132 (44.6%)	
Atrial fibrillation bradycardia	11 (2.6%)	1 (1.3%)	1 (1.9%)	9 (3.0%)	
Left ventricular ejection fraction, %	63.5 (60.4, 66.7)	64.7 (60.6, 67.1)	63.2 (61.0, 67.8)	63.3 (60.3, 66.3)	.27
<50%	27 (6.4%)	4 (5.3%)	5 (9.4%)	18 (6.1%)	.61
eGFR, mL/min/1.73m^2^	51.4 (39.1, 64.8)	54.3 (43.4, 69.9)	51.0 (45.0, 61.7)	50.6 (38.4, 64.5)	.18
BNP, pg/dL	142.6 (67.1, 413.0)	205.7 (70.3, 466.7)	103.8 (45.2, 218.5)	153.0 (69.7, 444.3)	.017
Medication at discharge					
Antiplatelet therapy	142 (33.5%)	21 (28.0%)	17 (32.1%)	104 (35.1%)	.49
Anticoagulants	132 (31.1%)	16 (21.3%)	14 (26.4%)	102 (34.5%)	.066
β‐blockers	89 (21.0%)	15 (20.0%)	9 (17.0%)	65 (22.0%)	.70
ACEI/ARB	208 (49.1%)	37 (49.3%)	27 (50.9%)	144 (48.6%)	.95
Diuretics	82 (19.3%)	15 (20.0%)	8 (15.1%)	59 (19.9%)	.71
SGLT2 inhibitors	14 (3.3%)	2 (2.7%)	4 (7.5%)	8 (2.7%)	.18
Mineralocorticoid receptor antagonist	49 (11.6%)	10 (13.3%)	7 (13.2%)	32 (10.8%)	.77
Antiarrhythmic drugs	34 (8.0%)	3 (4.0%)	4 (7.5%)	27 (9.1%)	.34

*Note*: Values are median (lower and upper qualities) or number (percentage).

Abbreviations: ACEI, angiotensin‐converting enzyme inhibitor; ARB, angiotensin receptor blocker; BMI, body mass index; BNP, brain natriuretic peptide; CABG, coronary artery bypass graft; HBP, His bundle pacing; LBBAP, left bundle branch area pacing; PCI, percutaneous coronary intervention; RVAP, right ventricular apical pacing; SGLT2, sodium–glucose cotransporter 2.

### 3.3 Procedural characteristics and results

Table 2 describes procedural characteristics and results. Dual‐chamber implantation was performed in 97% of patients. The HBP group required a longer procedural time than the LBBAP and RVAP groups (69.0 min [60.0, 86.0] vs. 65.0 min [56.0, 81.0] vs. 64.0 min [51.5, 78.0], *p* = .017). Procedural complications (e.g., cardiac effusion, lead revision, and lead extraction) rarely occurred in all groups. The delta QRS duration before and after the procedure increased in the HBP and RVAP groups, whereas it decreased in the LBBAP (13.0 ms [3.0, 33.0] vs. 40.0 ms [14.0, 66.0] vs. 0.0 ms [−19.0, 18.0], *p* < .001). V6RWPT was 68 ms (62.0, 76.0) in the LBBAP group, 82 ms (73.0, 94.0) in the HBP group. The HBP group had a higher capture threshold and a lower R wave amplitude than the LBBAP and RVAP groups (1.2 V [0.90, 1.70] vs. 0.90 V [0.70, 1.00] vs. 0.70 V [0.50, 0.90], *p* < .001; 3.5 mV [2.7, 4.8] vs. 9.5 mV [7.7, 13.8] vs. 7.8 mV [5.5, 10.5], *p* < .001, respectively). At the follow‐up, the median ventricular pacing rate was significantly higher in the LBBAP group than in the HBP and RVAP groups (98.7% [49.5, 99.9] vs. 61.8% [6.2, 99.6] vs. 15.9% [4.0, 96.6], *p* < .001). Capture threshold increase >1V during the follow‐up did not occur in the LBBAP group, although it was observed in 9.4% and 5.1% of the HBP and RVAP groups, respectively (*p* = .013). Nine cases (17.0%) of the HBP group were classified as selective HBP. Of the 75 cases of the LBBAP group, 60 (80.0%) were classified as LBBP. V6RWPT was 65 ms [61.0, 70.5]. The remaining 15 cases (20.0%) were classified as LVSP, with V6RWPT of 83 ms [78.5, 90.5]. Paced QRS duration had a short selective HBP of 96 ms [89.0, 109.0] and a relatively short LBBP of 114 ms [108, 126].

**TABLE 2:** Procedural and pacing characteristics.

	Overall (*n* = 424)	LBBAP (*n* = 75)	HBP (*n* = 53)	RVAP (*n* = 296)	*p* Value
Procedural time, min	65.0 (54.0, 80.0)	65.0 (56.0, 81.0)	69.0 (60.0, 86.0)	64.0 (51.5, 78.0)	.017
Fluoroscopy time, min	12.0 (8.0, 16.0)	12.0 (8.0, 16.5)	13.0 (8.0, 17.0)	11.0 (8.0, 16.0)	.093
Measurement at implantation					
Intrinsic QRS duration, ms	104 (88, 136)	124 (91, 140)	94 (88, 112)	104 (88, 138)	.006
Paced QRS duration, ms	146 (124, 162)	117 (110, 128)	124 (108, 134)	158 (146, 168)	<.001
Delta QRS duration, ms	24.0 (2.0, 54.0)	0.0 (−19.0, 18.0)	13.0 (3.0, 33.0)	40.0 (14.0, 66.0)	<.001
V6RWPT, ms	NA	68.0 (62.0, 76.0)	82.0 (73.0, 94.0)	NA	<.001
Capture threshold, V	0.80 (0.60, 1.00)	0.90 (0.70, 1.00)	1.20 (0.90, 1.70)	0.70 (0.50, 0.90)	<.001
R wave amplitude, mV	7.8 (5.2,10.6)	9.5 (7.7, 13.8)	3.5 (2.7, 4.8)	7.8 (5.5, 10.5)	<.001
Ventricular impedance, Ohms	678 (585, 799)	817 (710, 885)	551 (490, 616)	659 (585, 778)	<.001
Complications associated with procedure					
Cardiac effusion	4 (0.9%)	0 (0.0%)	1 (1.9%)	3 (1.0%)	.54
Lead revision	5 (1.2%)	1 (1.3%)	1 (1.9%)	3 (1.0%)	.86
Lead extraction	1 (0.2%)	1 (1.3%)	0 (0.0%)	0 (0.0%)	.097
Measurement at last follow‐up					
Ventricular pacing rate, %	32.4 (5.8, 99.1)	98.7 (49.5, 99.9)	61.8 (6.2, 99.6)	15.9 (4.0, 96.6)	<.001
>20%	235 (55.4%)	64 (85.3%)	32 (60.3%)	139 (47.0%)	<.001
Capture threshold, V	0.80 (0.60, 1.00)	0.75 (0.70, 1.00)	1.10 (0.90, 2.00)	0.80 (0.60, 1.00)	<.001
R wave amplitude, mV	10.3 (6.1, 16.0)	20.0 (15.7, 20.0)	4.6 (2.7, 7.2)	9.5 (6.3, 13.9)	<.001
Ventricular impedance, Ohms	494 (399, 570)	532 (494, 589)	418 (380, 456)	494 (399, 585)	<.001
Change in threshold, V	0.1 (−0.2, 0.3)	−0.05 (−0.2, 0.2)	0.0 (0.3, 0.5)	0.1 (−0.1, 0.4)	.013
>1V	20 (4.7%)	0 (0.0%)	5 (9.4%)	15 (5.1%)	.04

*Note*: Values are median (lower and upper qualities) or number (percentage). Abbreviations as in Table 1.

### 3.4 Clinical outcomes

**FIGURE 2:**
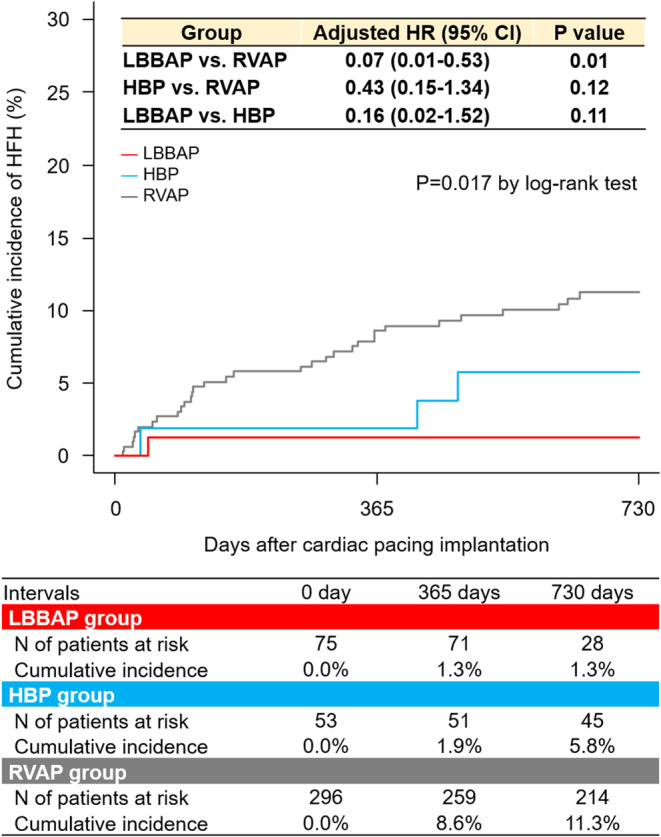
Cumulative 2‐year incidence of heart failure hospitalization. Abbreviations as in Table 1.

**FIGURE 3:**
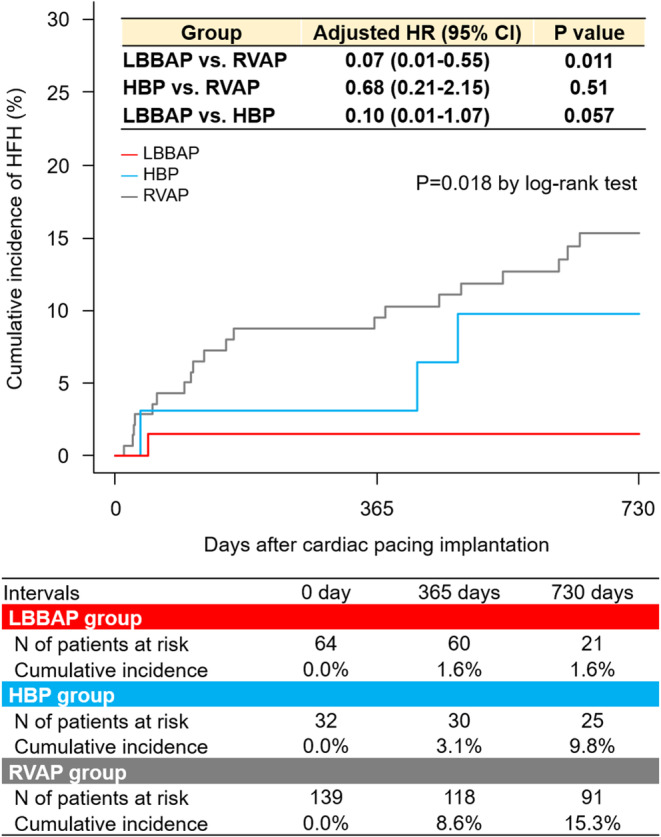
Cumulative 2‐year incidence of heart failure hospitalization in patients with ventricular pacing >20%. Abbreviations as in Table 1.

The overall median follow‐up was 2.9 years (2.0 to 3.6 years) (LBBAP, 1.9 years [1.6 to 2.1 years]; HBP, 3.1 years [2.7 to 3.5 years]; and RVAP, 3.2 years [2.4 to 3.8 years]). Table 3 summarizes clinical events during the entire follow‐up. HFH occurred only in one patient (1.3%) of the LBBAP group over the 2‐year follow‐up, although it did in 5.8% and 11.3% of the HBP and RVAP groups, respectively (Table 3 and Figure 2). The LBBAP group showed a lower incidence of HFH than the RVAP group (adjusted HR, 0.07 [95% CI: 0.01–0.53]; *p* = .01), whereas no significant difference was observed between the HBP and RVAP groups (adjusted HR, 0.43 [95% CI: 0.15–1.34]; *p* = .12) (Table 3). HFH occurred less frequently in the LBBAP group than in the HBP group, but it did not reach statistical significance (adjusted HR, 0.16 [95% CI: 0.02–1.52]; *p* = .11) (Table 3). Of 60 HFH cases, 16 (RVAP, *n* = 15; HBP, *n* = 1) (26.7%) were attributed to PICM. The cumulative incidence of MACE did not significantly differ between the 3 groups (*p* = .11) (Table 3). Cardiac death and BVP upgrade were observed only in the RVAP group (Table 3). Among patients with %RV pacing >20%, the LBBAP group also showed a lower incidence of HFH than the RVAP group (adjusted HR, 0.07 [95% CI: 0.01–0.55]; *p* = .011), whereas no significant difference was observed between the HBP and RVAP groups (adjusted HR, 0.68 [95% CI: 0.21–2.15]; *p* = .51) (Figure 3). HFH occurred less frequently in the LBBAP group than in the HBP group (adjusted HR, 0.10 [95% CI: 0.01–1.07]; *p* = .057) (Figure 3). The cumulative incidence of HFH was significantly lower in the CSP group than in the RVAP group (adjusted HR, 0.30 [95% CI: 0.12–0.76]; *p* = .01) (Figure S1).

**TABLE 3:** Clinical events during the follow‐up.

Outcomes	Patients with events (Cumulative 2‐year incidence)			Overall *p* value	LBBAP vs. RVAP		HBP vs. RVAP		LBBAP vs. HBP	
	LBBAP (*n* = 75)	HBP (*n* = 53)	RVAP (*n* = 296)		Adjusted HR (95% CI)^a^	*p* Value	Adjusted HR (95% CI)^a^	*p* Value	Adjusted HR (95% CI)^a^	*p* Value
HFH	1 (1.3%)	4 (5.8%)	45 (11.3%)	.017	0.07 (0.01–0.53)	.01	0.43 (0.15–1.25)	.12	0.16 (0.02–1.52)	.11
MACE	5 (8.0%)	9 (13.2%)	66 (16.5%)	.11	0.29 (0.11–0.75)	.01	0.63 (0.0–1.31)	.21	0.46 (0.15–1.42)	.18
All‐cause death	4 (6.6%)	6 (9.4%)	30 (7.3%)	.88	0.79 (0.25–2.49)	.68	1.04 (0.41–2.62)	.94	0.76 (0.20–2.92)	.69
Cardiac death	0 (0.0%)	0 (0.0%)	9 (2.3%)	.18	NA	NA	NA	NA	NA	NA
BVP upgrading	0 (0.0%)	0 (0.0%)	3 (1.0%)	.52	NA	NA	NA	NA	NA	NA

*Note*: Values are number (%), unless otherwise indicated. The number of patients with event was counted until the end of follow‐up. The cumulative 2‐year incidence was estimated by the Kaplan–Meier method. HRs with 95% CIs of (1) the LBBAP group relative to the RVAP and HBP groups and (2) the HBP group relative to the RVAP group for the outcome measures were estimated throughout the entire follow‐up period by the Cox proportional hazard models.

^a^ Adjusted for the following variables: age, male gender, body mass index, atrioventricular block, hypertension, dyslipidemia, diabetes mellitus, left ventricular ejection fraction <50%, hemodialysis, prior arterial fibrillation, prior coronary artery graft bypass, prior percutaneous coronary intervention, prior heart failure hospitalization, and percent right ventricular pacing.

Abbreviations: BVP, biventricular pacing; CI, confidence interval; HFH, heart failure hospitalization; HR, hazard ratio; MACE, major cardiovascular events; NA, not applicable. Other abbreviations as in Table 1.

### 3.5 Factors associated with HFH

Risk factors associated with HFH included age (HR, 1.04 [95% CI: 1.00–1.07]; *p* = .042), mean %RV pacing (per 10% increase) (HR, 1.12 [95% CI: 1.04–1.20]; *p* = .002), and LVEF <50% (HR, 2.52 [95% CI: 1.06–5.96]; *p* = .036) (Table 4). Compared to RVAP, LBBAP was negatively associated with HFH (HR, 0.08 [95% CI: 0.01–0.63]; *p* = .016) (Table 4).

**TABLE 4:** Factors associated with heart failure hospitalization.

Variables	Crude HR (95% CI)	*p* Value	Adjusted HR (95% CI)	*p* Value
Pacing mode (vs. RVAP)				
HBP	0.48 (0.17–1.34)	.16	0.41 (0.15–1.17)	.09
LBBAP	0.12 (0.02–0.86)	.035	0.08 (0.01–0.63)	.016
Mean %RV pacing (per 10% increase)	1.09 (1.02–1.16)	.014	1.12 (1.04–1.20)	.002
Left ventricular ejection fraction <50%	2.14 (0.91–5.03)	.08	2.52 (1.06–5.96)	.036
Age (per 1 year increase)	1.04 (1.01–1.08)	.018	1.04 (1.00–1.07)	.042
Prior atrial fibrillation	1.20 (0.64–2.21)	.57	1.45 (0.76–2.78)	.26

Abbreviation: %RV, percent right ventricular. Other abbreviations as in Table 1.

## 4 DISCUSSION

The main findings of the present study can be summarized as follows: (1) the LBBAP group showed a high procedural success rate without cardiac threshold increase >1V during the follow‐up; (2) the cumulative incidence of HFH was significantly lower in the LBBAP group than the RVAP group, while it did not differ between the HBP and RVAP groups; (3) HFH tended to occur less frequently in the LBBAP group than in the HBP group, although without statistical significance; (4) PICM did not occur in the HBP and LBBAP groups; and (5) advanced age, mean %RV pacing (per 10% increase), LVEF <50%, and RVAP (vs. LBBAP) were associated with HFH.

### 4.1 Comparison of technical aspects between HBP and LBBAP

His bundle pacing is a physiological alternative to RVAP with improved clinical outcomes.^4^ However, successful HBP with an adequate capture threshold is more technically challenging than RVAP because of the much smaller potential target area for lead, so its procedural and fluoroscopy times are prolonged compared to the RVAP.^4^ Consistently, the present study demonstrated that the HBP group had longer procedural and fluoroscopy times than the RVAP group. Although further improvement in devices and techniques might mitigate these limitations, it seems impossible to make procedural and fluoroscopy times for HBP shorter than those for RVAP. In contrast, LBBAP has an obvious advantage over HBP in terms of technical aspects as follows: (1) the site of pacing can be positioned distal to the pathological or vulnerable region within the conduction system, and (2) it is easier to perform LBBAP than HBP owing to the reduced requirement for precision needed in lead placement.^6^ In the present study, the procedural and fluoroscopy times were comparable between the LBBAP and RVAP groups. Also, the technical success rate was higher in the LBBAP group than in the HBP group (94.9% vs. 81.0%), in line with previous studies.^7^, ^26^, ^27^ Given these results, LBBAP might be more feasible than HBP from a technical viewpoint.

### 4.2 Complications associated with physiological pacing

Most complications associated with cardiac pacing occur in the periprocedural phase, but a sizable risk remains during the long‐term follow‐up.^28^ Recent studies have raised concern regarding HBP capture threshold increase during the long‐term follow‐up, which resulted in pacing failure and shorter battery longevity.^5^, ^28^, ^29^, ^30^ Vijayaraman et al.^29^ reported that His capture threshold increase >1V was noted in 12% of patients during 5‐year follow‐up; the need for lead revisions and generator change was higher in the HBP group than in the RVAP group (6.7% vs. 3.0% and 9.0% vs. 1.0%, respectively). In LBBAP, the lead is positioned slightly distal to the His bundle and is screwed deep within the left ventricular septum.^6^ These features lead to excellent results of electronic parameters (e.g., narrow QRS duration and stable pacing threshold). However, data on the long‐term lead performance of LBBAP are still scarce. The current study demonstrated that threshold elevation >1V occurred in the HBP and RVAP groups, while it did not in the LBBAP group. Although these results suggested the advantage of LBBAP over HBP in real‐world practice, further studies are warranted to assess the long‐term safety and efficacy of LBBAP because of the shorter follow‐up duration and relatively small study population.

### 4.3 Clinical outcomes after physiological pacing

Chronic RV pacing potentially leads to dyssynchronous ventricular activation and subsequent LV dysfunction (i.e., PICM); PICM occurs in ≈12% of patients with chronic RV pacing, although with a significant range between studies.^31^ Physiological pacing such as HBP and LBBAP is a novel technique directly activating the specialized conduction system and has reduced all‐cause mortality and HFH compared to RVAP.^4^, ^11^, ^12^, ^30^, ^31^ A recent meta‐analysis reported that pacing parameters (capture threshold and R wave amplitude) might be better in LBBAP than in HBP.^32^ To date, however, there is little evidence comparing clinical outcomes between HBP and LBBAP. The present study demonstrated that (1) the LBBAP group showed a significantly lower incidence of HFH than the RVAP group, while the HBP group did not; (2) PICM contributed to 26.7% of HFH cases, although without any PICM cases in the HBP and LBBAP groups. Notably, in the present study, the cumulative incidence of HFH was not significantly lower in the HBP group than in the RVAP group; most HFH occurred in the HBP group beyond 1‐year after implantation. These results might be explained by inherent limitations of HBP (e.g., HBP capture threshold increase in the chronic phase). In the current study, pacing failure attributable to an increased capture threshold constituted 50% of HFH cases in the HBP group. Furthermore, 17% of the HBP group comprised selective HBP, potentially leading to an elevated capture threshold when compared to the LBBAP group. Although these findings might contribute to no significant differences in HFH occurrence between the HBP and RVAP groups, it is prudent to interpret our results with caution due to the relatively limited sample size of the HBP group. Yet, it is noteworthy that PICM did not occur in the HBP and LBBAP groups. Also, the CSP group had a lower incidence of HFH than in the RVAP group, which was in line with the previous study.^10^ These results highlighted the clinical advantages of physiological pacing over RVAP.

### 4.4 Risk factors associated with HFH

Previous studies reported that baseline LV function, native QRS duration, RV pacing percentage, and paced QRS duration were primarily associated with the development of PICM.^1^, ^31^ A mode selection trial reported that the cumulative percent RV pacing strongly predicted HFH in patients with sinus node dysfunction.^1^ Consistently, the current study demonstrated that mean %RV pacing contributed significantly to the occurrence of HFH. Notably, despite the higher mean RV pacing rate than other groups, the LBBAP group had a protective impact on HFH compared with RVAP. Also, LVEF <50% was a risk factor for HFH in the current study, which was in line with previous studies.^33^ A current guideline strongly recommends BVP in HF patients with LVEF ≤35%, QRS duration ≥150 ms, and LBBB irrespective of optimal medical treatment.^28^ However, a recent randomized controlled trial reported the superiority of LBBAP over BVP in LVEF improvement in patients with nonischemic cardiomyopathy and LBBB.^31^ Although these results should be interpreted with caution because of the small study population, LBBAP might emerge as a first‐line approach in patients requiring cardiac pacing in the future, regardless of the baseline LV function. Further larger‐scale, randomized studies are warranted to establish the optimal management of those patients.

### 4.5 Limitations

The present study has several limitations. First, this study was a retrospective study; therefore, the sample size could not be calculated. Although we performed multivariable Cox models to account for variations in baseline clinical characteristics across the three groups, the inherent potential for bias in this study is inevitable, which might influence the drawn conclusions. Second, the selection of the pacing system was left to operator's discretion. Also, HBP and LBBAP have been available since June 2018 and November 2019, respectively. Thus, the follow‐up period of the HBP and LBBAP groups was shorter than the RVAP group. These findings might have biased the conclusions in the present study. Third, echocardiography was performed in 60.6% of cases during the follow‐up, which might result in the underestimation of PICM incidence. Fourth, the current study included both selective and nonselective HBP cases. Fifth, we confirmed right bundle branch block in the V1 lead and short V6RWPT in the LBBAP group, while not recording the left bundle branch potential. Finally, extrapolation of our results outside Japan requires caution because this study population consisted solely of Japanese subjects.

## 5 CONCLUSIONS

The procedural success rate was higher in the LBBAP group than in the HBP group without cardiac threshold increase >1V during the follow‐up. HFH occurred less frequently in the LBBAP group than the RVAP and HBP groups, although without a statistical significance between the LBBAP and HBP groups. Our results suggested that LBBAP appeared more feasible and effective in patients with bradycardia requiring permanent cardiac pacing.

## CONFLICT OF INTEREST STATEMENT

All authors have no relevant financial or nonfinancial interests to disclose.

## ETHICS APPROVAL

The research protocol received approval from the ethics committee at Kokura Memorial Hospital and adhered to the principles outlined in the Declaration of Helsinki.

## CONSENT TO PARTICIPATE

Not applicable (Written informed consent was waived because of the retrospective study design).

## CLINICAL TRIAL REGISTRATION

This study was registered with http://www.umin.ac.jp, unique identifier UMIN000053252.

## Supporting information


Figure S1.

## Data Availability

The data that support the findings of this study are available from the corresponding author upon reasonable request.
