# Supplementary material for: Outcomes of left bundle branch area pacing compared to His bundle pacing and right ventricular apical pacing in Japanese patients with bradycardia
Source: J Arrhythm. 2024 Jan 28;40(2):333–41. doi: 10.1002/joa3.12997 (PMC10995588; doi:10.1002/joa3.12997)
Supplement: Supplementary file 1 — Figure S1. [file JOA3-40-333-s001.docx]

**Supplementary Figure 1: Cumulative Incidence of Heart Failure Hospitalization in Patients with Conduction System Pacing and Right Ventricular Apex Pacing**


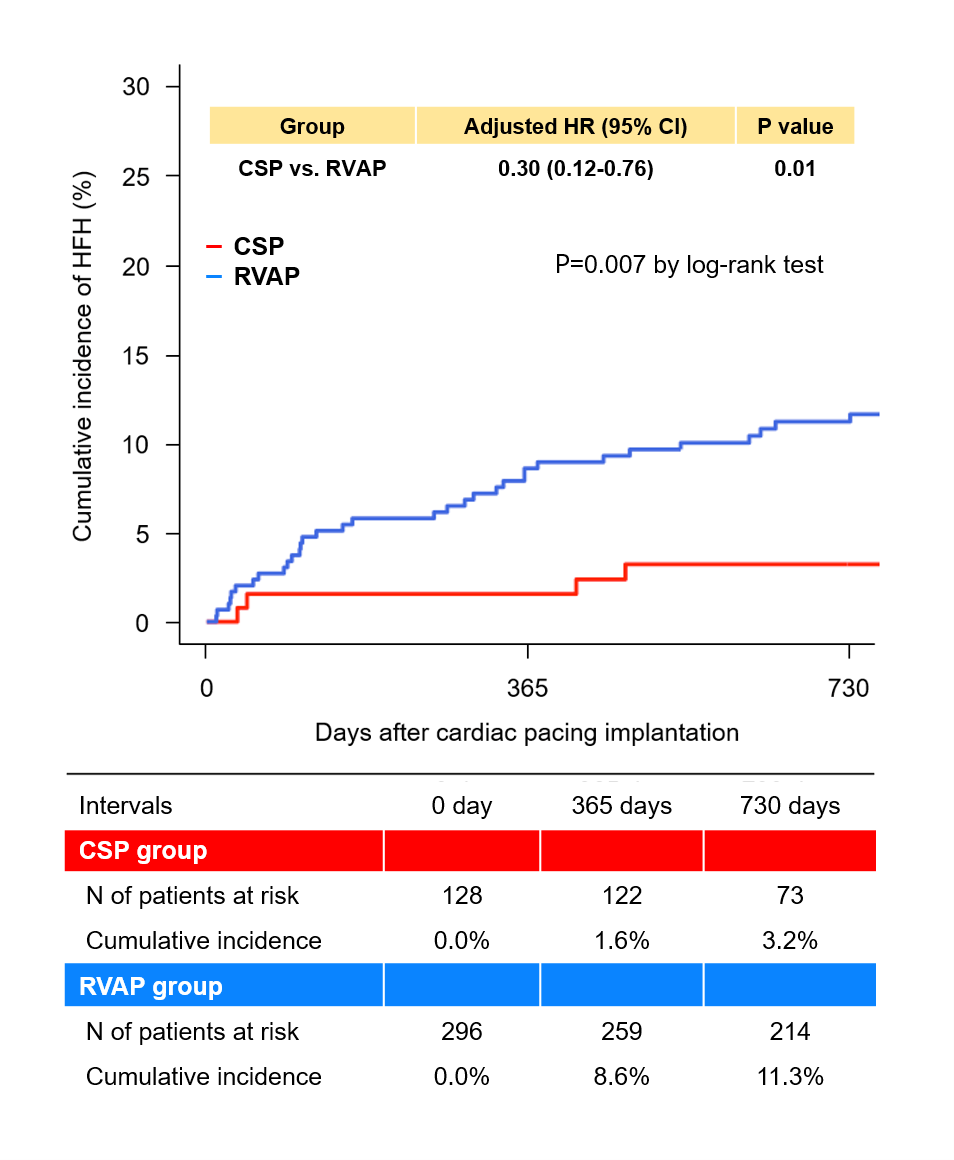


CI = confidence interval; CSP = conduction system pacing; HFH = heart failure hospitalization; RVAP = right ventricular apex pacing
